# Combined score based on plasma fibrinogen and platelet-lymphocyte ratio as a prognostic biomarker in esophageal squamous cell carcinoma

**DOI:** 10.1186/s12885-024-11968-6

**Published:** 2024-02-22

**Authors:** Yuchong Yang, Hui Tan, Yao Lu, Jipeng Mei, Mengqi Zhang, Ming Bai, Xia Wang, Shaohua Ge, Tao Ning, Le Zhang, Zhi Ji, Jingjing Duan, Yansha Sun, Feixue Wang, Rui Liu, Hongli Li, Ting Deng

**Affiliations:** 1 Department of GI Medical Oncology, Tianjin Medical University Cancer Institute and Hospital, National Clinical Research Center for Cancer, Tianjin’s Clinical Research Center for Cancer, Tianjin Key Laboratory of Digestive Cancer, Key Laboratory of Cancer Prevention and Therapy, Huanhuxi Road, Tiyuanbei, Hexi District, 300060 Tianjin, China; 2 Department of Surgical Oncology and General Surgery, The First Affiliated Hospital of China Medical University, Shenyang, Liaoning China; 3 Tianjin University of Traditional Chinese Medicine, Tianjin, China

**Keywords:** Esophageal squamous cell carcinoma, Fibrinogen- platelet to lymphocyte ratio score, Neutrophil to lymphocyte ratio, Platelet to lymphocyte ratio, Prognosis

## Abstract

**Background:**

Increasing evidence has showed that inflammatory biomarkers, including neutrophil to lymphocyte ratio (NLR), platelet to lymphocyte ratio (PLR) and fibrinogen can be used as predictors in the prognosis of esophageal squamous cell carcinoma (ESCC). The aim of this study was to explore prognostic value of these biomarkers and evaluate the clinicopathological and prognostic significance of combined score based on plasma fibrinogen and platelet-lymphocyte ratio (F-PLR score).

**Methods:**

A total of 506 patients with ESCC were enrolled in this study. Harrell’s concordance index (c-index) was used to determine the optimal cut-off values of these markers and evaluate their prognostic significance. The relationship between factors with survival rates (including overall survival [OS] and disease-free survival [DFS]) was explored by Kaplan-Meier curve, univariate analysis and multivariate cox hazard analysis.

**Results:**

Our result indicated that high F-PLR score was significantly associated with longer tumor length and deeper depth of tumor invasion (*p* < 0.01). The result of Cox multivariable analysis showed that F-PLR score was an independent prognostic factor for OS (*p* = 0.002) and DFS (*p* = 0.003). In addition, F-PLR score presented the greater c-index values for OS and DFS compared with NLR, PLR and fibrinogen level. Our result also showed that the c-index values for OS and DFS were both greater in TNM + F-PLR than those in TNM stage alone.

**Conclusions:**

In conclusion, F-PLR score is a predictive biomarker for prognosis in patients with ESCC.

**Supplementary Information:**

The online version contains supplementary material available at 10.1186/s12885-024-11968-6.

## Background

Esophageal cancer is one of the most aggressive cancers in gastrointestinal tract cancers and it is the fourth leading cause of cancer death in China [1, 2]. Esophageal squamous cell carcinoma (ESCC) is the major histological type of esophageal cancer in Asian countries, including Japan and China [3]. Although great improvement has been made in the diagnosis and treatment of ESCC in recent years, the clinical outcome of patients still remains poor [4]. Therefore, identifying a simple and dependable biomarker that could distinguish ESCC patients with high risk of tumor progression and poor prognosis is extremely urgent.

Growing evidence has showed the tumor progression and prognosis are determined not only by features of tumor but also host inflammation response [5, 6]. Recently, evidence has increasingly shown that the inflammatory biomarkers including neutrophil to lymphocyte ratio (NLR), platelet to lymphocyte ratio (PLR) has been reported to be a prognostic predictor in cancers [7, 8], including ESCC [9–11]. On the other hand, fibrinogen is a pro-inflammatory protein synthesized by hepatocytes and converted to fibrin in response to infection, tissue injury, or inflammation [12, 13], and it has been reported to play an important role in the coagulation cascade, which takes a key role in the process of tumor progression and metastasis [14]. In addition, some studies reported that plasma fibrinogen levels were significantly associated with tumor development and prognosis in several types of cancers [15, 16], including ESCC [17, 18]. Previous study by Yang et al. has demonstrated that PLR was a superior prognostic predictor compared with NLR in ESCC patients [19]. Therefore, we proposed a novel prognostic biomarker based on a combination of plasma fibrinogen levels and PLR in patients with cancers. Until now, the clinical value of the combined score of fibrinogen levels and PLR (F-PLR score) in blood specimens from ESCC patients has not been reported.

The aim of this present study was to explore the association of the F-PLR score and clinicopathological features. We also assessed the prognostic significance of these biomarkers (including F-PLR score, NLR, PLR and plasma fibrinogen) and compared their prognostic capacity in ESCC.

## Materials and methods

### Patients

We reviewed the data of ESCC patients who underwent radical surgery at the Tianjin Medical University Cancer Institute and Hospital between May 2005 and December 2021. The criteria for patient selection were as follows: (1) Pathologic diagnosis of primary ESCC; (2) no history of preoperative adjuvant therapy; (3) preoperative blood routine result and fibrinogen levels obtained within 2 weeks before operation; (4) no history of neoadjuvant therapy or preoperative anti-inflammatory treatment, or long-term use of anticoagulant drugs. Patients with multiple cancers and patients who received palliative resection were excluded in our study. The following information was recorded for all ESCC patients: age, gender, operation date, tumor location, max tumor diameter, tumor differentiation, TNM stage, depth of tumor invasion, status of lymph node metastasis and preoperative laboratory data (including neutrophil, lymphocyte and platelet counts in routine blood test and fibrinogen levels in blood clotting function test). Patients were classified and staged based on the 8th edition of the AJCC/UICC tumor-node-metastasis (TNM) classification system. All ESCC patients were followed-up every 3 to 6 months after operation by regular clinical examinations, such as tumor marker tests (CEA, SCC, Cyfra21-1), ultrasonography, and computed tomography. The median follow-up period was 44 months (range, 2-107 months).

### Statistical analysis

Association between the fibrinogen levels and the PLR (F-PLR) score and clinicopathological features were assessed using the Chi-squared test. Survival rates, including overall survival (OS) and disease-free survival (DFS), were assessed using Kaplan–Meier method with the log-rank tests. Univariate analysis was used to determine the association between the prognostic factors and survival rates. Significant prognostic factors for survival rates in univariate analysis were included in the multivariate analyses using the Cox proportional hazards model with input method.

We assessed the predictive prognostic ability of different classification by measuring discrimination, which is the ability to distinguish between high-risk and low-risk patients. The method of Harrell’s concordance index (c-index) was used to evaluated the discrimination and the optimal cut-off values of biomarkers [20, 21]. The maximum c-index value was 1.0 and a higher c-index indicated a more desirable model for predicting the prognosis.

Statistical analyses were performed using SPSS software version 22.0 (SPSS, Chicago, IL, USA) and STATA software (version 18.0, Stata Corporation, College Station, TX, USA). All statistical analyses were two-sided, and a *p*-value of less than 0.05 was considered statistically significant.

## Result

### Optimal cut-off values of biomarkers

Until now, controversy still exists on the optimal cut-off values of NLR, PLR and fibrinogen for predicting prognosis in ESCC. In our study, we use the method of c-index to determine the optimal cut-off values of these biomarkers. The results showed that the c-index values were maximum for OS (and/or DFS) when NLR, PLR and fibrinogen were 1.6, 126, and 3.8, respectively (Table S1). For further analysis, we separated ESCC patients into two groups (NLR < 1.6 and ≥ 1.6; PLR < 126 and ≥ 126; fibrinogen < 3.8 g/L and ≥ 3.8 g/L).

### Clinicopathological factors and F-PLR score

The F-PLR score was divided into three groups based on each cut-off value of fibrinogen and PLR as follows; F-PLR score of 2: with high fibrinogen level (≥ 3.8 g/L) and high PLR (≥ 126), F-PLR score of 1: with one of these hematological abnormalities, and F-PLR score of 0: with neither high fibrinogen level nor high PLR. We enrolled 506 patients with ESCC in this study, including 413 (81.0%) males and 93 (19.0%) females. The median age of these patients was 61 years (range, 33–92). The patients’ baseline characteristics and clinicopathological features divided by F-PLR score were described in Table 1.

**Table 1:** Relationship between clinicopathological features and F-PLR score in ESCC

Variable	F-PLR score			
	0(%)	1(%)	2(%)	*p*
Age(y)				0.345
<60	91 (46.9)	83 (39.7)	44 (42.7)	
≥60	103 (53.1)	126 (60.3)	59 (57.3)	
Gender				0.791
Male	159 (82.0)	168 (80.4)	86 (83.5)	
Female	35 (18.0)	41 (19.6)	17 (16.5)	
Tumor location				0.207
Upper	26 (8.0)	12 (6.6)	38 (7.5)	
Middle	200 (61.7)	101 (55.5)	301 (59.5)	
Lower	98 (30.2)	69 (37.9)	167 (33.3)	
Tumor length (cm)				**< 0.001**
<3.5	94 (48.5)	73 (34.9)	24 (23.3)	
≥3.5	100 (51.5)	136 (65.1)	79 (76.7)	
Differentiation				0.804
Well - moderate	150 (77.3)	163 (78.0)	83 (80.6)	
Poor	44 (22.7)	46 (22.0)	20 (19.4)	
Depth of tumor invasion				**0.001**
T1-2	82 (42.3)	67 (32.1)	22 (21.4)	
T3-4	112 (57.7)	142 (67.9)	81 (78.6)	
Lymph node metastasis				0.074
N0	114 (58.8)	101 (48.3)	60 (58.3)	
N1-3	80 (41.2)	108 (51.7)	43 (41.7)	
TNM stage				0.152
I-II	82 (42.3)	69 (33.0)	37 (35.9)	
III-IV	112 (57.7)	140 (67.0)	66 (64.1)	
Adjuvant chemotherapy				0.789
Yes	79 (40.7)	90 (43.1)	46 (44.7)	
No	115 (59.3)	119 (56.9)	57 (55.3)	

Abbreviations, F-PLR score: fibrinogen and platelet-lymphocyte ratio score

Our result indicated that the high F-PLR score was significantly associated with longer tumor length and deeper depth of tumor invasion (*p* < 0.01, Table 1). There was no significant association between F-PLR score and age, gender, tumor location, tumor differentiation, lymph node metastasis, TNM stage, status of adjuvant chemotherapy (*p* > 0.05, Table 1).

### Prognosis and F-PLR score

**Fig. 1:**
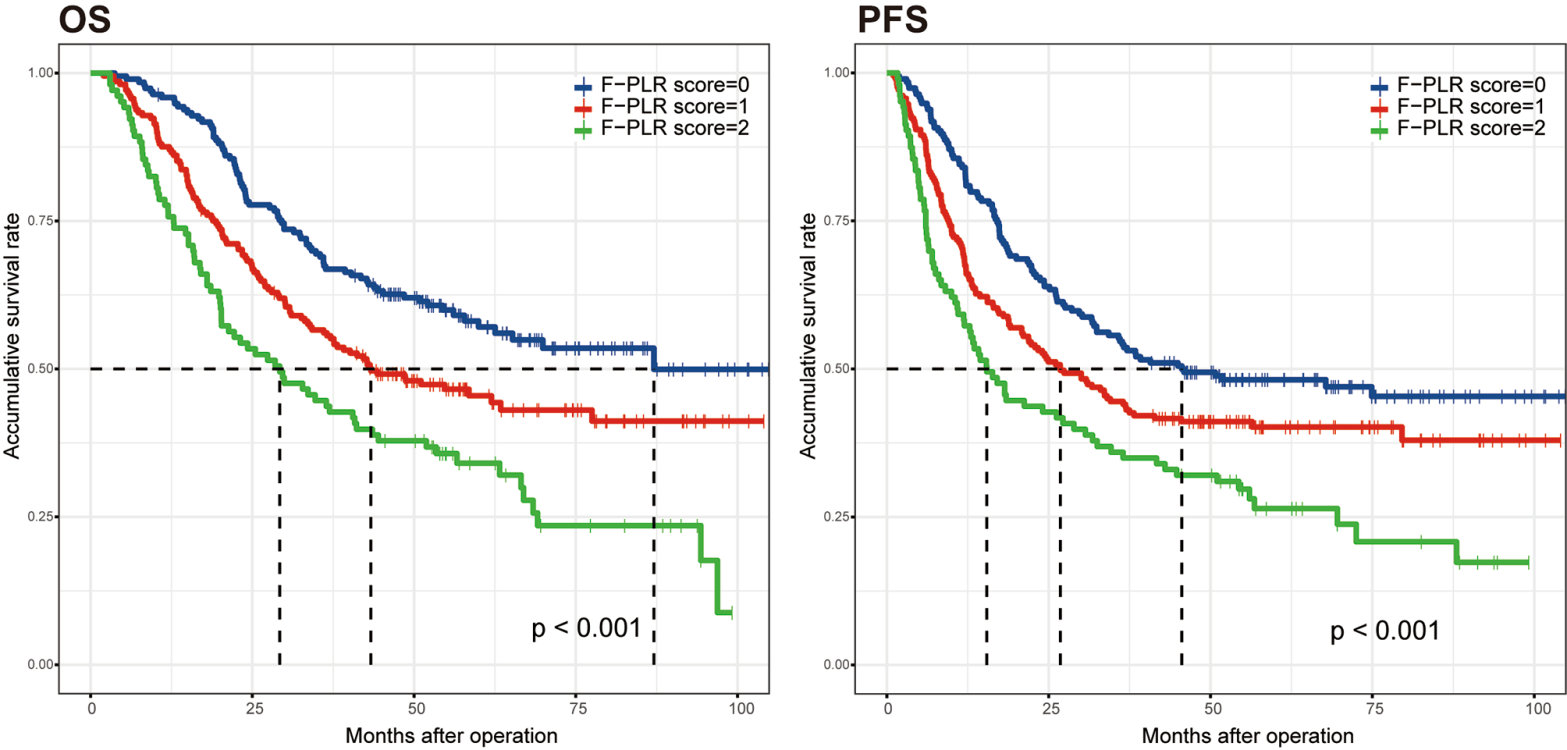
Kaplan-Meier curves of overall survival and disease-free survival in ESCC patients based on F-PLR score

In our study, the methods of Kaplan-Meier with log-rank tests and univariate analysis were used to explore the association of prognosis and biomarkers. The result of Kaplan-Meier curve indicated that high NLR, high PLR, high plasma fibrinogen level and high F-PLR score were significantly associated with poor OS and DFS (*p* < 0.05, Fig. 1, Figure S1).

The result of univariate analysis showed that longer tumor length, depth of tumor invasion, lymph node metastasis, TNM stage, NLR, PLR, fibrinogen level and F-PLR score were significantly associated with OS and DFS (*p* < 0.05, Table 2). Moreover, Cox multivariable analysis showed that F-PLR score was an independent prognostic factor for OS (*p* = 0.002) and DFS (*p* = 0.003, Table 2).

**Table 2:** Univariate and multivariate survival analyses of OS and DFS in patients with ESCC

Variable	Overall survival				Disease free survival			
	Univariate		Multivariate		Univariate		Multivariate	
	HR (95% CI)	*p*	HR (95% CI)	*p*	HR (95% CI)	*p*	HR (95% CI)	*p*
Age (y)		0.613				0.577		
<60	1				1			
≥60	1.064 (0.836–1.355)				0.938 (0.748–1.176)			
Gender		0.071				0.293		
Male	1				1			
Female	0.736 (0.528–1.026)				0.852 (0.632–1.148)			
Tumor length (cm)		**< 0.001**		**0.042**		**< 0.001**		0.069
<3.5	1		1		1		1	
≥3.5	1.980 (1.518–2.583)		1.345 (1.010–1.791)		1.844 (1.442–2.358)		1.276 (0.981–1.659)	
Differentiation		0.982				0.305		
Well - moderate	1				1			
Poor	1.003 (0.750–1.342)				1.150 (0.880–1.502)			
Depth of tumor invasion		**< 0.001**		**< 0.001**		**< 0.001**		**< 0.001**
T1-2	1		1		1		1	
T3-4	3.005 (2.208–4.088)		2.364 (1.707–3.275)		2.446 (1.865–3.206)		1.838 (1.378–2.450)	
Lymph node metastasis		**< 0.001**		**< 0.001**		**< 0.001**		**< 0.001**
N0	1		1		1		1	
N1-3	2.450 (1.917–3.130)		2.468 (1.901–3.205)		2.355 (1.872–2.964)		2.056 (1.624–2.602)	
TNM stage		**< 0.001**				**< 0.001**		
I-II	1				1			
III	3.752 (2.756–5.109)				3.264 (2.480–4.295)			
Adjuvant chemotherapy		**0.010**		**< 0.001**		0.108		
No	1		1		1			
Yes	0.731 (0.575–0.928)		0.476 (0.370–0.612)		0.831 (0.663–1.042)			
NLR		**0.010**		0.532		**0.034**		0.886
<1.6	1		1		1		1	
≥1.6	1.454 (1.095–1.931)		1.103 (0.812–1.497)		1.325 (1.021–1.720)		1.021 (0.770–1.354)	
PLR		**< 0.001**				**0.001**		
<126	1				1			
≥126	1.619 (1.275–2.058)				1.462 (1.167–1.831)			
Fibrinogen (g/L)		**< 0.001**				**0.001**		
<3.8	1				1			
≥3.8	1.655 (1.301–2.107)				1.489 (1.184–1.873)			
F-PLR score		**< 0.001**		**0.002**		**< 0.001**		**0.003**
0	1		1		1		1	
1	1.513 (1.140–2.009)		1.248 (0.931–1.673)		1.336 (1.029–1.736)		1.165 (0.888–1.529)	
2	2.327 (1.700-3.184)		1.725 (1.222–2.434)		1.967 (1.463–2.646)		1.639 (1.183–2.270)	

Abbreviations, CI: confidence interval; F-PLR score: fibrinogen and platelet-lymphocyte ratio score; HR: hazard ratio; NLR: neutrophil to lymphocyte ratio; PLR: platelet to lymphocyte ratio

### Prognostic ability of F-PLR score

**Fig. 2:**
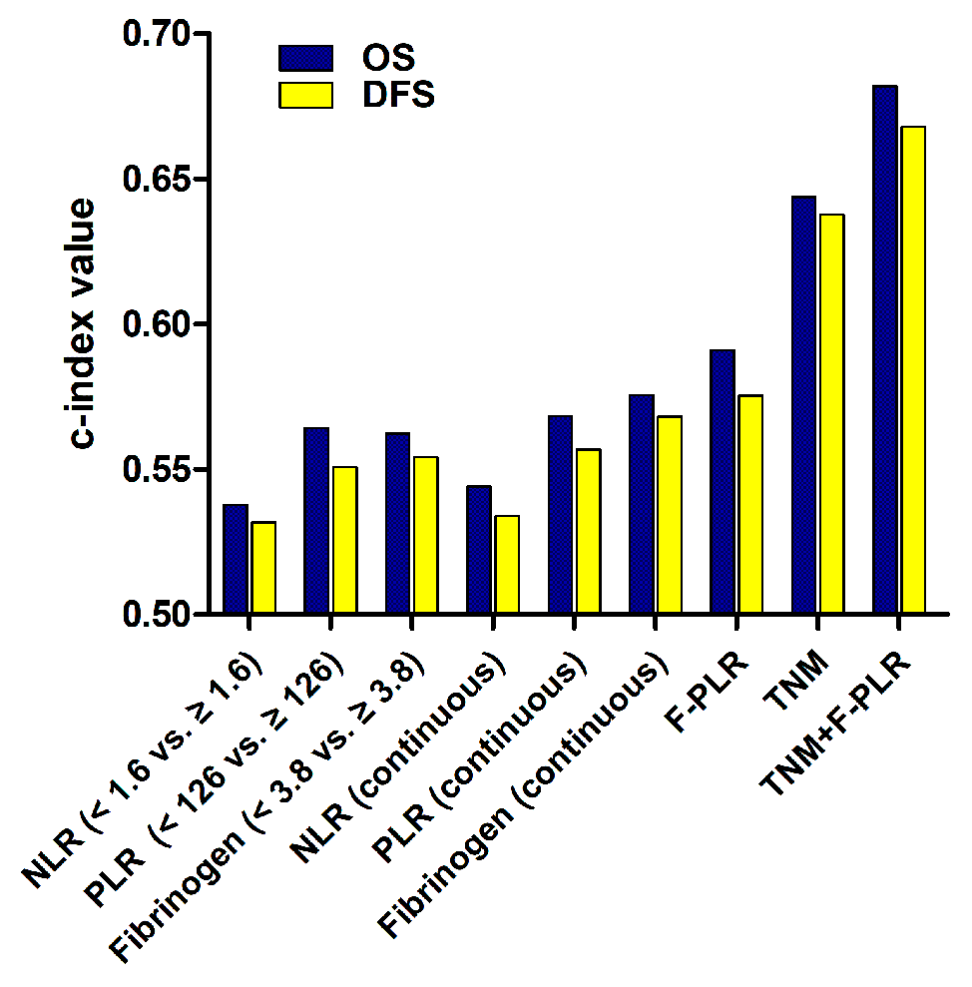
Comparison of the c-index values for biomarkers on overall survival and disease-free survival

In this study, we used the method of c-index to compare the prognostic value of these biomarkers. The result indicated that F-PLR score presented the maximum c-index value for OS and DFS compared with NLR, PLR and fibrinogen level (no matter whether they were regarded as dichotomous variables or continuous variables). On the other hand, we also calculated the c-index value of TNM stage combined with F-PLR score (TNM + F-PLR) and of TNM stage alone for OS and DFS. Our result demonstrated that the c-index values were greater (OS: 0682 vs. 0.644; DFS: 0.668 vs. 0.638) in TNM + F-PLR than those in TNM stage alone (Fig. 2).

## Discussion

In this study, we retrospectively reviewed a cohort of ESCC patients in our institution. Our result indicated that high F-PLR score was significantly related with advanced tumor features, including longer tumor length and deeper depth of tumor invasion. On the other hand, the result of Kaplan-Meier with log-rank tests and univariate analysis both showed that high F-PLR score were significantly associated with poor OS and DFS. In addition, Cox multivariable analysis indicated that F-PLR score was an independent prognostic factor in ESCC. These results demonstrated that F-PLR score was a predictor of prognosis and was significantly associated with advanced tumor features in ESCC patients. To the best of our knowledge, this is the first report to study the clinicopathological and prognostic value of F-PLR score in patients with ESCC.

There were several reasons to explain the association of F-PLR score and poor prognosis. First, Platelets and fibrinogen together plays a key role in cancer progression (including growth, invasion and metastasis) by promoting cancer neovascularization and supporting the sustained adhesion of cancer cells [22, 23], Palumbo et al. found that platelet-fibrin (ogen) axis may increase metastatic potential by impeding natural killer cell-mediated elimination of cancer cells [24]. While lymphocytes are the important components of the immune system and can kill cancer cells and prevent cancer progression [25]. Thrombocytosis, hyperfibrinogenemia and low lymphocyte count were proved to be significantly associated with poor prognosis in different types of cancer [26–29]. Therefore, this may partly explain why high F-PLR score was significantly related with poor prognosis in ESCC. On the other hand, the result showed that high F-PLR score was significantly related with advanced tumor features. Therefore, high F-PLR score may be associated with the extent of cancer progression and consequently may affect the prognosis of ESCC patients. However, we should note that cancers usually induce a hypercoagulable state in the host and may results in thrombocytosis and hyperfibrinogenemia [30]. Whether high F-PLR score is a cause or consequence of cancer progression still remains unknown and needs to be confirmed in the future.

Based on previous reports, we hypothesized that the combined score of fibrinogen levels and PLR these two markers may be a better indicator for prognosis in ESCC at the beginning of the study. The results bear out our supposition. In this study, the method of c-index was used to compare the prognostic capacity of these biomarkers. Our result indicated that F-PLR score presented the greater c-index values for OS and DFS compared with NLR, PLR and fibrinogen. In addition, we found that the c-index values for OS and DFS were greater in TNM + F-PLR than those in TNM stage alone, indicating that TNM + F-PLR had better prognostic ability than TNM stage alone. Therefore, F-PLR score may provide an additional biomarker in the current TNM stage system and increase the prognostic accuracy in ESCC.

Until now, there is no uniform method to determine an optimal cut-off value of these biomarkers suitable for all patient cohorts. In our study, c-index, which is a commonly used method to evaluate the predictive prognostic capacity of models, was used to explore the optimal cut-off value among these ESCC patients. However, whether this cut-off value determined by the patient cohort in our institution can be applied to other independent patient cohorts needs to be further confirmed.

There are several limitations in our study. First, the data of these patients with ESCC was reviewed retrospectively and all patients are from only one single institution. Second, because of the lack of relevant data, we did not explore the clinicopathological and prognostic value of other inflammatory biomarkers, such as acute-phase proteins and procalcitonin. Third, we did not analyze the impact of comorbidities (diabetes, coronary heart disease, hypertension, thromboembolic disease et al.) or other unmeasured variables on the F-PLR score and patient outcomes due to the lack of relevant information.

## Conclusions

In summary, F-PLR score has clinical potential as a predictive biomarker for prognosis in patients with ESCC.

### Electronic supplementary material

Below is the link to the electronic supplementary material.

Supplementary Material 1

## Data Availability

The datasets used during the current study are available from the corresponding author on reasonable request.
