# Supplementary material for: Combined score based on plasma fibrinogen and platelet-lymphocyte ratio as a prognostic biomarker in esophageal squamous cell carcinoma
Source: BMC Cancer. 2024 Feb 22;24:249. doi: 10.1186/s12885-024-11968-6 (PMC10885567; doi:10.1186/s12885-024-11968-6)
Supplement: Supplementary file 1 — Supplementary Material 1 [file 12885_2024_11968_MOESM1_ESM.docx]

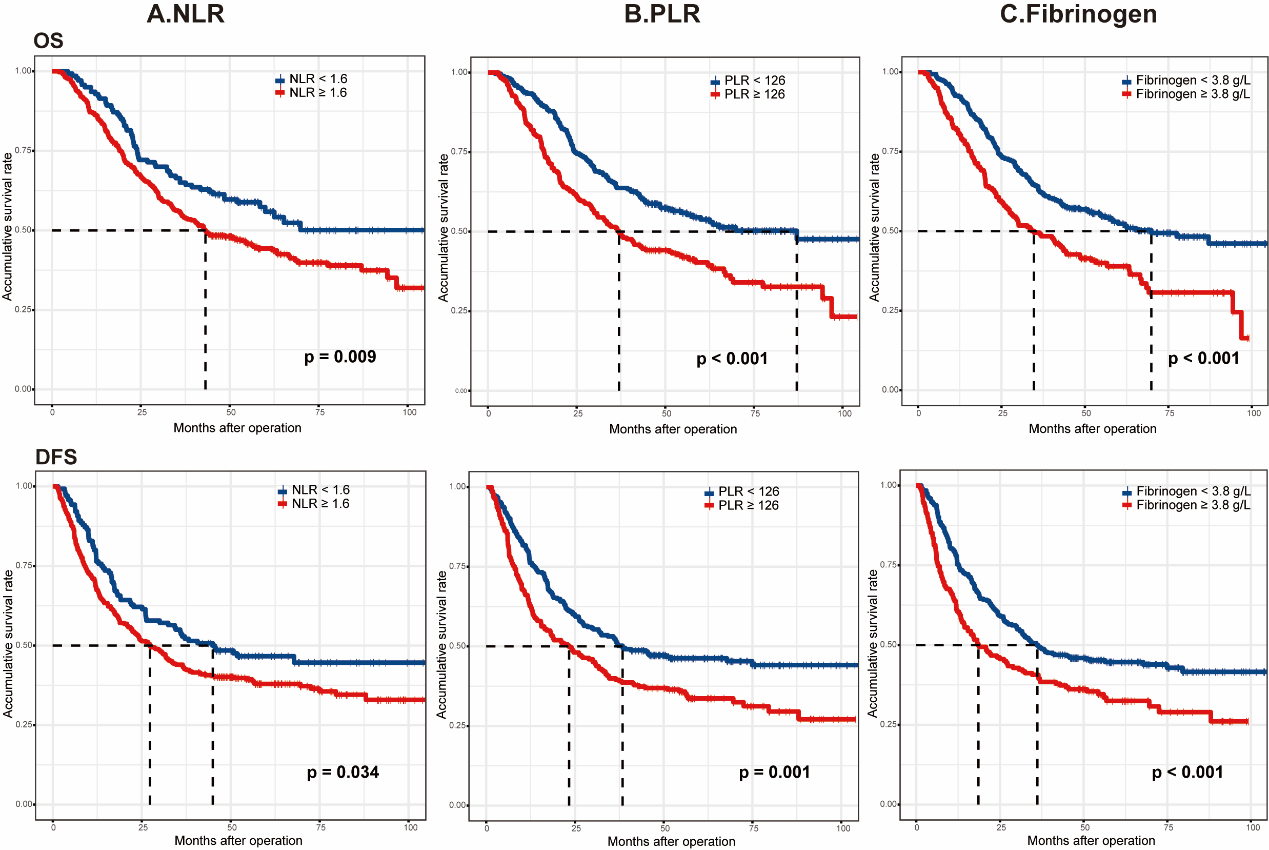


**Figure S1.** Kaplan-Meier curves of overall survival and disease-free survival in ESCC patients based on biomarkers: (A) NLR; (B) PLR; (C) Fibrinogen

**Table S1** The five greatest c-index values of different cut-off for NLR, PLR and fibrinogen

| Survival | NLR | | |  | PLR | | |  | Fibrinogen | | |
| --- | --- | --- | --- | --- | --- | --- | --- | --- | --- | --- | --- |
|  | Cut-off | c-index | N |  | Cut-off | c-index | N |  | Cut-off | c-index | N |
| OS | 1.6 | 0.5378 | 140/366 |  | 126 | 0.5644 | 273/233 |  | 3.8 | 0.5623 | 324/182 |
|  | 1.9 | 0.5377 | 224/282 |  | 129 | 0.5643 | 294/212 |  | 3.6 | 0.5569 | 293/213 |
|  | 1.8 | 0.5367 | 195/311 |  | 128 | 0.5643 | 288/218 |  | 3.7 | 0.5566 | 313/193 |
|  | 1.2 | 0.5265 | 53/453 |  | 124 | 0.5641 | 270/236 |  | 3.9 | 0.5528 | 362/144 |
|  | 1.7 | 0.5344 | 168/338 |  | 132 | 0.5613 | 308/198 |  | 4.1 | 0.5473 | 396/110 |
| DFS | 1.6 | 0.5319 | 140/366 |  | 118 | 0.5531 | 238/268 |  | 3.8 | 0.5541 | 324/182 |
|  | 1.8 | 0.5291 | 195/311 |  | 126 | 0.5508 | 273/233 |  | 3.9 | 0.5506 | 362/144 |
|  | 1.9 | 0.5281 | 224/282 |  | 132 | 0.5505 | 308/198 |  | 3.7 | 0.5497 | 313/193 |
|  | 1.7 | 0.5271 | 168/338 |  | 124 | 0.5499 | 270/236 |  | 4.0 | 0.5492 | 381/125 |
|  | 1.3 | 0.5270 | 67/439 |  | 129 | 0.5503 | 294/212 |  | 4.1 | 0.5473 | 396/110 |

Abbreviations, DFS: Disease free survival; N: number of patients for each group; NLR: neutrophil to lymphocyte ratio; OS: overall survival; PLR: platelet to lymphocyte ratio
